# It Doesn’t Look Like Work, but it Works: A Qualitative Study of Staff Experiences with a Self-Harm Strategy in Danish Inpatient Mental Health Services

**DOI:** 10.1007/s10488-026-01506-w

**Published:** 2026-04-27

**Authors:** Kristine Høst Poulsen, Lisbeth Hybholt, Carsten Hjorthøj, Erik Simonsen, Bo Bach, Philip Tønder Hartvig, Mickey Toftkjær Kongerslev

**Affiliations:** 1 Mental Health Services East, Region Zealand, Roskilde, Denmark; 2 Department of Psychology, University of Southern Denmark, Odense, Denmark; 3 Department of Regional Health Research, University of Southern Denmark, Odense, Denmark; 4 Copenhagen Research Unit for Mental Health – CORE, Copenhagen University Hospital, Copenhagen, Denmark; 5 Department of Public Health, Section of Epidemiology, University of Copenhagen, Copenhagen, Denmark; 6 Department of Clinical Medicine, University of Copenhagen, Copenhagen, Denmark; 7 Mental Health Services West, Region Zealand, Slagelse, Denmark; 8 Department of Psychology, University of Copenhagen, Copenhagen, Denmark; 9 Child and Adolescent Mental Health Services, Region Zealand, Roskilde, Denmark

**Keywords:** Self-harm, NSSI, Mental health services, Inpatient care, Staff perspectives, Focus groups, Reflexive thematic analysis, Coercion, Implementation, Staff training

## Abstract

Non-suicidal self-injury (NSSI) is common in inpatient mental health care and poses challenges for staff, patients, and organizations. While training programs can improve knowledge and attitudes, less is known about how staff collectively experience implementing new strategies for managing self-harm and how such strategies influence ward culture and professional practice. This qualitative study was part of a broader evaluation of implementing a regional self-harm strategy in a Danish inpatient mental health service. The strategy included staff training, clinical tools, and supervision. Four focus group interviews with 25 staff members from five inpatient units were conducted in 2024. Data were analyzed using reflexive thematic analysis. The analysis generated four interrelated themes: (1) *Relational work as invisible yet essential*: Staff valued listening and dialogue as crucial for preventing escalation but described these practices as undervalued and the first to be dropped under pressure. (2) *Diagnostic hierarchies: psychosis as the legitimate inpatient diagnosis*,* self-harm as responsibility*: Psychotic patients were viewed as genuinely ill and beyond control, while patients who self-harm were often framed as responsible, sustaining moralized hierarchies of care. (3) *Cultural change – from control to dialogue*: The strategy disrupted routines of restraint in relation to self-harm. Initially perceived as “doing nothing,” the new strategy was increasingly legitimized as staff witnessed calmer units, reduced coercion, and clearer professional roles grounded in relational practice. (4) *Responsibility*,* guilt*,* and emotional distance* — Staff struggled with the emotional and moral burden of refraining from coercion, negotiating professional boundaries, and managing feelings of guilt and emotional withdrawal. Implementing a self-harm strategy requires more than new clinical tools. It depends on staff integrating the approach into everyday practice, supported by structural and cultural change. Sustainable implementation relies on legitimizing relational care as skilled clinical work, creating space for reflection, and addressing the emotional demands placed on staff. The findings also show that diagnostic hierarchies continue to shape how legitimacy and responsibility are distributed within psychiatric care.

**Supplementary Information:**

The online version contains supplementary material available at 10.1007/s10488-026-01506-w.

## Background

Self-harm is a pressing challenge in psychiatric inpatient care, closely linked to suicide risk (Bowers et al., 2010), repetition of self-harm episodes (Hawton et al., 2015), and psychological distress for patients, relatives, and staff (Carroll et al., 2014). Episodes of self-harm frequently escalate to coercive interventions and conflict (Groves et al., 2024), while staff report feelings of frustration, helplessness, and limited structural support when caring for people at risk of self-harm or suicide (Saunders et al., 2012; Boukouvalas et al., 2020). Patients, in turn, often encounter stigma and moralized responses that undermine therapeutic engagement (Chaney, 2017; Ring & Lawn, 2019). Recent research further underscores how suicide-related stigma contributes to defeat, entrapment, and social isolation, reinforcing distress and creating systemic barriers to compassionate care (Wyllie et al., 2025).

These challenges have led mental health systems internationally to explore strategies that strengthen relational and reflective approaches to care and reduce reliance on coercion. In this context, Region Zealand Mental Health Services developed a self-harm strategy aimed at shifting inpatient practice away from routine coercion toward dialogue, patient involvement, and reflective clinical engagement. The strategy emphasized understanding self-harm within its emotional and interpersonal functions and supporting patients as active participants in their care. This orientation aligns with principles of therapeutic risk-taking, which encourage informed autonomy in situations that involve degrees of risk (Felton et al., 2017). The development of the strategy reflects broader international policy trends that promote reduced coercion, strengthened relational care, and recovery-oriented approaches in mental health services.

In this paper, the term self-harm is used in the broad sense commonly applied in European and clinical policy contexts, referring to deliberate self-injury with or without suicidal intent. This usage aligns with how the Regional Self-Harm Strategy and participating staff understand the phenomenon in practice. In clinical practice, self-harm is typically used as an umbrella term that includes both suicidal and non-suicidal behaviors, and the distinction debated in the literature reflects conceptual and research-focused classifications rather than discrete clinical phenomena. Recent conceptual work also highlights ongoing efforts to clarify the construct of NSSI, including proposals to shift its designation from a standalone disorder to a clinical specifier, reflecting persistent uncertainty about how best to classify non-suicidal self-injury within psychiatric nosology (Lengel et al., 2022, 2025). The regional self-harm strategy was developed primarily to address NSSI, and where research or diagnostic frameworks use this term, this paper draws on that literature directly. However, in acute inpatient settings, staff encounter self-harm across a spectrum that includes NSSI, suicidal self-injury, and presentations that are difficult to classify in the moment — not least because patients may shift between functions over time, and because articulating suicidal intent clearly is itself a clinical skill that the strategy aims to develop. This paper therefore uses self-harm as an umbrella term throughout, while drawing on NSSI literature where relevant and noting the distinction where it matters for the analysis.

## Stigma, Moralization, and Professional Responses

Contemporary research highlights NSSI as a coping strategy serving affect-regulatory and interpersonal functions (Klonsky, 2007). Yet despite strong empirical evidence framing NSSI as a symptom of emotion dysregulation rather than volition (Kongerslev et al., 2015; Jørgensen et al., 2024), psychiatry has historically interpreted it in moralizing terms, often linking it to borderline personality disorder and perpetuating stigma (Chaney, 2017; Ring & Lawn, 2019). This association is partly rooted in the diagnostic criteria for borderline personality disorder, where non-suicidal self-injury appears as one of the defining features. These legacies continue to shape staff perceptions and responses in inpatient settings.

Although NSSI is strongly associated with borderline personality disorder, with around half of those who engage in NSSI meeting diagnostic criteria for BPD (Andrews et al., 2019; Goodman et al., 2017; Zanarini et al., 2006), it also occurs across a wide range of other psychiatric diagnoses. Recent population-based and clinical studies also highlight autistic spectrum conditions as an important context for self-harm, underscoring both the heterogeneity of self-injuring behaviors and the challenges of underdiagnosis in acute psychiatric settings (Stark et al., 2022; Kim et al., 2024; Kildahl et al., 2025). NSSI is now recognized as a transdiagnostic phenomenon and is included as a condition for further study in DSM-5 (American Psychiatric Association, 2013). Research also shows that self-harm occurs in individuals without psychiatric diagnoses, highlighting broader psychosocial and developmental determinants (Møhl, 2019). This dual positioning, both closely associated with BPD yet by no means confined to it, helps explain why stigma toward BPD remains central for understanding professional responses to self-harm. Longitudinal research further demonstrates that self-harm often persists despite treatment, challenging notions of it as volitional or manipulative behavior (Jørgensen et al., 2024).

While training interventions have been shown to improve knowledge and attitudes (Saunders et al., 2012; Rayner et al., 2019), meta-analytic evidence indicates that stigmatizing attitudes remain common, especially among nurses in emergency and inpatient settings (Rayner et al., 2019). Recent studies confirm that negative and moralizing attitudes persist despite increased attention to education and guidelines (Uddin et al., 2024; Lantto et al., 2025), particularly in acute psychiatric and emergency care where staff often report emotional strain and limited structural support.

Much of this research has focused on individual outcomes, such as changes in knowledge, confidence, or attitudes among staff, leaving less understood about how staff collectively experience change in everyday practice, and how the implementation of a self-harm strategy may shape professional culture and influence staff understandings and approaches to self-harm within inpatient settings.

A further aspect complicating the matter concerns the nature of relational work, listening, being present, and talking with patients. These practices are central to recovery (Leamy et al., 2011), yet they are often less visible and more difficult to measure than medical or procedural tasks (Groves et al., 2024; Hult et al., 2024). This “invisibility” of relational work reflects broader findings in mental health nursing research, where relational and narrative work is emotionally demanding yet structurally marginalized, a pattern reinforced by organizational valuation systems that privilege measurable activities over affective or dialogical practices (Johansson et al., 2010; Timmermans & Berg, 2003). More generally, this body of research shows how efficiency demands, standardization, and digital infrastructures constrain relational work by privileging procedural and measurable activity over dialogical or affective practices. Hagen et al. (2017) and Li et al. (2023) similarly emphasize the emotional strain and lack of organizational support surrounding self-harm care, while Grant et al. (2015) illustrate how institutional logics often devalue these dimensions.

Finally, not all patient groups are positioned equally. Borderline personality disorder is frequently stigmatized, perceived as difficult to treat, and associated with negative professional attitudes (Linehan, 1993; Kongerslev et al., 2015; Ring & Lawn, 2019). Although schizophrenia is heavily stigmatized in the general population, often due to perceptions of dangerousness (Clement et al., 2015), borderline personality disorder tends to elicit stronger negative reactions within mental health services themselves. Studies show that clinicians often attribute intentionality, controllability, or limited treatability to BPD, leading to more moralized and punitive responses than those directed toward other severe mental illnesses (Aviram et al., 2006; Knaak et al., 2015; Sheehan et al., 2016). Recent research shows that insufficient knowledge about BPD is closely linked to stigmatizing attitudes, including assumptions about volition, intentionality, and treatability (Marmin et al., 2025). Consistent with this, a qualitative study from China found that psychiatric nurses caring for patients with repeated non-suicidal self-injury often moved from empathy to frustration and resentment, describing the behavior as intentional or manipulative and responding with criticism or punitive measures (Yue et al., 2024). These moralized interpretations of repeated NSSI mirror findings in Western contexts, suggesting that negative attitudes toward patients who self-harm are a cross-cultural challenge. Such patterns not only affect patients but also staff well-being, since stigma and discrimination have been associated with burnout and compassion fatigue among mental health professionals (Del Olmo-Romero et al., 2025).

Taken together, existing research shows that self-harm in inpatient settings is shaped by stigma, moralization, professional hierarchies, and the structural marginalization of relational work. While many studies have focused on individual attitudes or competencies, far less is known about how staff collectively understand self-harm and how new strategies may influence professional culture and everyday practice. Examining these cultural and relational dimensions is essential for understanding how self-harm initiatives are experienced in inpatient care and for identifying the conditions that support or constrain meaningful change. Understanding such processes of collective change can be informed by Lewin’s (1947) model of organizational change, which describes how established norms are disrupted, new practices tested, and a revised understanding gradually stabilized — a framework that informs the interpretive lens of this study. The aim of this study was to explore how staff in acute psychiatric inpatient units experienced the implementation of a regional self-harm strategy, identifying factors that facilitated or hindered its delivery and understanding its emotional and professional impacts.

## Methods

### Study Design and Setting

This qualitative study was part of a broader project examining the implementation of a regional self-harm strategy developed and introduced by Region Zealand Mental Health Services, Denmark. A qualitative design was chosen to explore collective meaning-making among staff and to capture the cultural and emotional dimensions of practice that are not accessible through quantitative measures. According to Danish law, studies involving staff participants do not require approval from a research ethics committee. The study was conducted in accordance with institutional guidelines and the Declaration of Helsinki. All participants provided oral informed consent prior to participation. The regional self-harm strategy was mandated by Mental Health Services, Region Zealand, which consists of three administrative divisions (East, West, and South). This study was conducted in the Eastern division, where all five acute adult inpatient wards were selected for targeted implementation. Staff on all five wards received structured training, clinical tools, and regular supervision to support the integration of the strategy into everyday practice. Acute inpatient units in Denmark typically admit patients with psychotic, affective, and personality-related crises, and self-harm occurs across diagnostic groups. The five units together had a total capacity of 81 beds. Most units had approximately 13 beds, although capacity varied somewhat between units. One unit functioned as a double unit at the time of the study, comprising two 13-bed Sect.  (2 × 13 beds). In addition, the psychiatric emergency unit had 12 beds, including 8 open beds and 4 closed beds in the high-observation area.

### Self-Harm Strategy

The strategy was developed by an expert panel comprising clinicians, representatives from the police and municipalities, and a person with lived experience of self-harm. It aimed to strengthen knowledge, collaboration, and practice related to the management of self-harm across all inpatient mental health services. A central and innovative element of the strategy was the shift away from using coercive measures to stop self-harm, unless it posed an immediate risk to life. Staff were instead encouraged to tolerate limited self-harming behavior and focus on dialogue, validation, and de-escalation. This represented a significant departure from previous practice and required staff to adopt new relational and ethical approaches to safety and care.

The educational and supervisory components were developed and delivered by the researcher. A person with lived experience contributed to the training development by reviewing the initial teaching materials, providing feedback on content and wording, and suggesting adjustments that were incorporated into the final version of the training.

The training consisted of a one-day (five-hour) interactive teaching session for all ward staff, followed by a two-hour brush-up session approximately two months later. The sessions were based on principles from Dialectical Behavior Therapy (DBT) and included teaching on (a) the functions and meanings of self-harm, (b) emotional regulation and validation, (c) relational and organizational responses to self-harm, and (d) staff experiences and emotional reactions.

Two clinical tools were introduced to support everyday practice: (1) a pros and cons worksheet adapted from DBT, and (2) a pocket card summarizing key principles for communication and de-escalation. Additionally, two key staff members were appointed on each unit to receive ongoing supervision and additional training from the researcher.

The regional strategy also included a freely accessible e-learning module on self-harm for all staff, and regular group supervision sessions led by the researcher. In total, 188 staff members (98% of total staff) across the five units participated in the initial training between May and June 2023. The qualitative component of this study consisted of four focus group interviews conducted between May and June 2024. Further details about the regional strategy and its educational materials are available on the Region Zealand website (https://www.psykiatrienregsj.dk/om-psykiatrien/for-fagpersoner/faellesstrategi-for-haandtering-af-selvskade).

### Participants and Recruitment

Participants were inpatient staff employed at one of the five adult mental health inpatient units in Region Zealand Mental Health Services East involved in the implementation of the self-harm strategy. Staff were eligible if they had participated in the regional training program on self-harm and had at least three months of experience working with patients who engage in self-harm. Recruitment was coordinated by the researcher, who distributed study information via email to staff working on the inpatient wards. Participation was voluntary, and oral informed consent was obtained from all participants.

Across four focus groups, a total of 25 staff members participated, with between three and eight participants in each group. Variation in group size is well established in focus group methodology. Smaller groups can enable more in-depth reflection on emotionally demanding topics, whereas larger groups can reveal a broader range of shared norms and divergent perspectives (Barbour, 2007; Halkier, 2016; Malterud, 2011; Kitzinger, 1995). Participants represented a wide range of professional backgrounds, including registered nurses (*n* = 4), health care assistants (*n* = 6), social educators (*n* = 3), nursing students (*n* = 2), physiotherapists (*n* = 1), doctors (*n* = 3), psychologists (*n* = 1), managers (*n* = 2), and a psychomotor therapist (*n* = 1). The majority were female (*n* = 17), and participants’ ages ranged from 24 to 61 years, with clinical experience ranging from 0.6 to 18 years. This variation in experience was considered an asset, because differences in knowledge, confidence, and exposure to self-harm are integral to the collective meaning-making that focus groups are designed to explore.

This professional and demographic diversity provided a broad foundation for exploring how staff across disciplines and levels of experience perceived and implemented the self-harm strategy in daily clinical practice. No invited staff declined participation or withdrew from the study after the interviews.

### Data Generation

Data were generated through focus group interviews conducted in May and June 2024. Focus groups were used because of their capacity to elicit collective meaning-making and to explore how shared experiences are negotiated in interaction. This method is particularly suited for examining professional practice, social norms, and emotionally complex care work (Kitzinger, 1995; Barbour, 2007; Malterud, 2011; Halkier, 2016).

The focus groups were facilitated by the researcher (female psychologist) with more than ten years of clinical experience in Mental Health Services. No prior therapeutic relationships existed between the interviewer and participants. Participants were informed that the facilitator had been involved in delivering elements of the regional training program, but that participation was voluntary and unrelated to employment evaluation. This information was intended to promote openness and minimize power asymmetry during discussions.

Each focus group lasted approximately 2.5 h and was held in a meeting room at the participants’ workplace. Interviews were facilitated by the researcher, with a co-researcher present to take field notes except in one group due to scheduling conflicts. A semi-structured interview guide was used, covering topics such as experiences with the training program, use of new tools and practices, perceived changes in ward culture, and challenges in working with self-harm. Questions were open-ended, and follow-up probes were used to encourage elaboration and dialogue.

All focus groups were audio-recorded and transcribed verbatim. Identifying information was removed during transcription to ensure confidentiality for staff participants, any mentions of patients during the interviews, and the institutions involved.

### Data Analysis

Data were analyzed using Reflexive Thematic Analysis (RTA) as described by Braun and Clarke (2006, 2019, 2020, 2022). This approach emphasizes flexibility, researcher subjectivity, and the active role of the analyst in generating, rather than discovering, themes. The analysis was primarily inductive and sought to capture both explicit meanings and underlying assumptions in participants’ accounts. In addition to RTA, Lewin’s (1947) three-stage model of organizational change — unfreezing, moving, and refreezing — was drawn upon as an interpretive lens for understanding the cultural and organizational dimensions of implementation.

The analytic process was iterative and reflexive. The researcher and a co-researcher familiarized themselves with the data by reading transcripts and listening to audio recordings, making detailed notes and analytic memos. The researcher then coded the data manually without the use of qualitative software, identifying meaning units across transcripts and grouping them into preliminary patterns and visual maps. These patterns were gradually developed into candidate themes through ongoing comparison, refinement, and engagement with the full dataset. A co-researcher contributed through reflexive dialogue to deepen interpretation rather than to achieve inter-coder reliability.

Data collection continued until no substantially new insights emerged across focus groups, consistent with the principle of data sufficiency. Transcripts were not returned to participants; credibility was instead fostered through reflexive dialogue, memo-writing, and peer discussion.

Qualitative rigor was ensured through iterative theme development, rich data extracts, and systematic documentation of analytic decisions. Dependability and confirmability were supported by reflexive journaling and peer debriefing, while transferability was facilitated through detailed contextual description. The study is reported in accordance with principles of transparent qualitative research (Tong et al., 2007), while analytic procedures followed the Reflexive Thematic Analysis approach as outlined by Braun and Clarke (2006, 2019, 2021).

### Reflexivity

Reflexivity was central throughout the research process. The researcher, a female psychologist with ten years of clinical experience in psychiatry, brought professional familiarity with the field, which supported sensitivity to staff perspectives but required continuous reflexive awareness of assumptions and potential bias. Reflexivity was supported through journaling, peer debriefing, and discussions with a co-researcher. A central reflexive consideration concerns the researcher’s dual role as designer and lead trainer of the staff education program, and as the lead researcher studying its implementation. The focus groups asked staff to reflect on two time points, the period following the strategy announcement and the period following the training, meaning participants reflected on both an organizational policy and a program the researcher had designed and delivered. This position can be understood as observant participation in Seim (2024) sense: engaged, practice-near research that carries both epistemic advantages, contextual competence and access to backstage logics, and recognized risks of over-identification and social desirability. To mitigate this, participants were informed that participation was voluntary, responses confidential, and the study entirely independent of employment evaluation. The researcher aimed to adopt a non-evaluative facilitation style, actively encouraging critical and ambivalent accounts. These dynamics were documented through reflexive memos and revisited during analysis. Researcher positionality is therefore treated here not only as a source of potential bias but as a constitutive feature of the research design (Koobak & Thapar-Björkert, 2014): one that shaped what became speakable in the data and required continuous reflexive attention throughout the analytic process.

## Results

The four focus groups included 25 participants representing a range of professional backgrounds. In addressing the study’s research question, the analysis generated four interrelated but analytically distinct themes: (1) Relational work as invisible yet essential, (2) Patient hierarchies, psychosis as legitimate psychiatry and self-harm as responsibility, (3) Cultural change from control to dialogue, and (4) Responsibility, guilt, and emotional distance. These themes reflect how staff made sense of their everyday work, including how they interpreted different forms of distress, responsibility, and legitimacy within inpatient care. They should therefore be understood as analytic interpretations of shared meaning rather than as diagnostic or structural categories. Together, the themes illustrate how staff experienced the implementation of the self-harm strategy as both challenging and legitimizing. While relational care was often difficult to sustain and to make visible, the strategy reconfigured practices, patient categories, professional culture, and staff’s emotional positioning. Across themes, staff identified both barriers and facilitators to change, ranging from moral hierarchies and emotional strain to legitimizing experiences of success and support. These experiences highlight the emotional and professional impacts of implementation, including the strain, uncertainty, and eventual sense of recognition that accompanied cultural transformation in practice.

## Theme 1: Relational Work as Invisible Yet Essential

Staff described listening, talking, and being present with patients as central to preventing escalation and managing self-harm. Staff described these relational practices as undervalued or invisible within the wider system, reflecting how they understood the organizational priorities that shaped their everyday work.

*“This thing of just sitting and talking with people. Or making plans. It doesn’t look like work*,* but it is actually what works.” (FG2)*.

This illustrates how staff recognized the therapeutic effect of relational presence while simultaneously struggling to have such work recognized as *real* clinical practice. Relational care produced tangible results but lacked institutional visibility because it did not fit existing metrics of productivity or intervention.

*“If you talk to people in your social circle*,* and they ask*,* what do you actually do at work? (…) Then it can be hard to explain that it is sitting and talking with patients.” (FG3)*.

Here, staff frame the invisibility of relational work as a broader cultural problem: when care does not involve observable action, it risks being misunderstood as inactivity, both by outsiders and by the organization itself.

The same invisibility made this work emotionally demanding.

*“But it requires huge relational work. Huge*,* huge relational work. And it wears you down sometimes.” (FG3)*.

This suggests that invisibility carries a double burden: staff expend emotional energy without institutional recognition or structural protection, making relational work simultaneously vital and depleting.

Under staffing pressure, relational work was often the first to disappear:

*“The first thing we drop when we’re short on staff is the talking – because documentation and meds have to be done.” (FG1)*.

This tension reveals how systemic priorities, documentation and medication, displace relational practices, reinforcing their marginality despite their effectiveness.

Over time, however, the strategy helped legitimize relational work by connecting it to measurable outcomes such as calmer inpatient units and reduced coercion. What had once “not looked like work” became evidence of skilled professional practice.

## Theme 2: Patient Hierarchies – Psychosis as Legitimate Psychiatry, Self-Harm as Responsibility

Staff consistently distinguished between patients with psychotic symptoms and those who engaged in self-harm, creating a hierarchy of legitimacy. These hierarchies reflect staff’s meaning-making about legitimacy rather than diagnostic categories and describe how certain forms of distress were positioned as more “real” or deserving of care.

Psychotic patients were described as genuinely ill and not responsible for their actions, which elicited empathy and intervention. In contrast, patients who self-harmed were often framed as exercising choice, and therefore as responsible for their distress.

*“When they are psychotic*,* they cannot help it. That is something completely different.” (FG4)*.

*“I have more understanding for a psychotic patient because they don’t know what they are doing. Someone with borderline knows what she is doing.” (FG3)*.

These quotes illustrate how psychiatric legitimacy was tied to perceived control and intention. When patients’ actions were seen as involuntary, staff could respond with compassion. When behavior appeared deliberate, empathy faltered, and responsibility was reassigned to the patient. This moral logic structured everyday care and positioned some forms of suffering as more “real” than others.

*“The new strategy… it brought so much uproar in the staff group. Suddenly it filled more than schizophrenia and that*,* which we usually think of as our core.” (FG1)*.

Here, staff articulate how these hierarchies defined psychiatry’s boundaries — what counted as “real” psychiatric work and what did not. The introduction of the self-harm strategy unsettled these boundaries by asking staff to take seriously a patient group historically viewed as outside their core mandate.

At the same time, some participants articulated counter-narratives that challenged this hierarchy:

*“We also have to remember that it is her way of handling pain.” (FG3)*.

*“I had to ask the patient herself what I should do. (…) It was a recognition that she had more experience in this situation than I did.” (FG1)*.

These reflections signal an emerging shift in professional understanding. By framing self-harm as a coping strategy rather than manipulation, staff began to see patients as collaborators in care rather than as moral agents to be corrected. This opened space for empathy and curiosity, enabling a more dialogical relationship.

Taken together, this theme highlights how moralized distinctions between “illness” and “choice” shaped the emotional landscape of care. They acted as barriers to implementing the new strategy because tolerance of self-harm initially conflicted with prevailing notions of professionalism. Yet, the emergence of alternative framings, self-harm as coping, the patient as expert, functioned as facilitators of change, allowing staff to align empathy with legitimacy.

## Theme 3: Cultural Change – from Control to Dialogue

The self-harm strategy represented a profound cultural shift in inpatient psychiatry. Staff contrasted earlier routines of control and restraint, where the main goal was to stop self-harm “at any cost”, with the new emphasis on dialogue, tolerance, and reflection.

*“We just had to restrain at any cost. (…) And I think back*,* wow. We could have avoided so many things if we had had the new strategy.” (FG1)*.

This recollection captures the taken-for-granted nature of earlier coercive practices. What once felt self-evident and morally necessary later appeared excessive or even harmful, illustrating how deeply the old culture of control had been internalized.

*“It feels strange to just let her do it. As if we don’t care.” (FG2)*.

Initially, many experienced the new approach as emotionally and ethically disorienting—*unfreezing* long-held norms about responsibility and care. Allowing limited self-harm without intervention challenged staff’s instinct to protect and their professional identity as “doers.” This discomfort signaled both the moral tension and the beginning of cultural movement.

*“I think we have experienced that the level of conflict goes down*,* because we take the dialogue instead.” (FG3)*.

*“Once we saw the figures*,* how much coercion had gone down*,* it clicked for me that she [the trainer] was right.” (FG1)*.

Over time, experiential learning and concrete results; calmer inpatient units, fewer coercive episodes, legitimized the new practice. Seeing measurable improvements enabled *refreezing* around new understandings of care: that safety could be maintained through conversation and trust rather than physical control. In this process, relational practices that had previously been perceived as passive or insufficient were gradually reframed as skilled and effective professional interventions. The implementation process also illustrates how emotional and structural dimensions of change were closely intertwined: staff reactions such as uncertainty, moral discomfort, and gradual acceptance were shaped not only by individual attitudes but by organizational conditions such as staffing levels, opportunities for reflection, and visible outcomes.

*“I believe we have less self-harm when there are enough staff on duty. (…) In the evenings*,* when there are fewer of us*,* it gets worse.” (FG1)*.

This observation highlights that cultural change depended not only on attitude but also on structure. Understaffing and time pressure undermined reflection and dialogue, revealing that organizational conditions either supported or eroded the sustainability of the new approach.

Together, these accounts illustrate a collective process of *unfreezing* established routines, *moving* through moral uncertainty, and *refreezing* around dialogue as a new professional norm. What began as resistance and confusion gradually became shared conviction that relational engagement, not restraint, constituted meaningful psychiatric work.

## Theme 4: Responsibility, Guilt, and Emotional Distance

Implementing the new self-harm strategy required staff to renegotiate what it meant to be responsible for patient safety. The shift from intervening to tolerating self-harm, unless it posed immediate danger, challenged ingrained instincts and professional norms.

*“I think the hardest thing has been to stand there and watch someone harm themselves*,* and not intervene*,* because it’s just ingrained in you that you must stop it. (…) That has been one of the most difficult things.” (FG3)*.

This quote captures the ethical dissonance of *doing less* to care more. Staff were asked to replace visible action with presence and dialogue, which felt at odds with long-standing ideals of protection and control.

*“When does it become fatal? (…) Is it me*,* as a nurse*,* who should decide that? (…) I don’t know if I think it’s my role to assess whether someone has cut too deep*,* if that’s where we end up.” (FG3)*.

Here, staff articulate the uncertainty that accompanied blurred professional boundaries. The strategy’s tolerance for non-intervention left individuals personally accountable for judging when to act, generating anxiety about moral and legal responsibility.

*“When you say relational work is tough*,* and it is. (…) But does anyone think about how much it actually takes from staff to give that little extra? (…) That also speaks to hardening*,* compassion fatigue*,* and burnout.” (FG3)*.

This suggests that the emotional labor of remaining empathic in the face of repeated self-harm was both essential and draining. Without explicit organizational recognition or collective structures for support, staff risked exhaustion and emotional withdrawal.

*“Some of us tried to work with the new strategy*,* but others sat with their arms crossed. (…) It could feel lonely to be the one who believed in it.” (FG2)*.

Moments like this reveal that change was not only intrapersonal but social. Implementing the new approach often meant standing apart from colleagues who resisted it, creating pockets of isolation amid cultural transformation.

Together, these narratives show how the new strategy redistributed responsibility from collective control to individual judgment and emotional resilience. Feelings of guilt (“Could I have done more?”), the need for distance, and sporadic collegial resistance exposed the personal cost of cultural change. Over time, however, ongoing reflection and dialogue allowed some staff to share this burden, marking the beginnings of a new collective ethic of care grounded in tolerance and mutual support.

## Discussion

This study examined how inpatient staff experienced the implementation of a regional self-harm strategy. The analysis showed that staff struggled with the invisibility of relational work, made sense of self-harm through moralized hierarchies, navigated a cultural shift from control to dialogue, and renegotiated responsibility in ways that created both emotional strain and professional growth.

The four themes highlight how the strategy reshaped staff’s understandings of their work, their patients, and the ward culture. This process can be viewed as paralleling Lewin’s (1947) description of organizational change, where established norms are unsettled, new practices are tested, and a different understanding of care gradually becomes stable in everyday work. Relational practices, long difficult to sustain and to make visible, became more acknowledged through their effects on conflict and coercion. Staff continued to negotiate patient hierarchies grounded in perceptions of legitimacy and responsibility, patterns that suggest the presence of an implicit diagnostic legitimacy hierarchy within inpatient psychiatry, in which different forms of distress were granted different degrees of professional recognition, while the shift from restraint to dialogue challenged established routines and moral expectations. The implementation of the strategy also placed new emotional demands on staff, who navigated uncertainty, strain, and moments of recognition as they made sense of their changing roles.

## Relational Work and Invisibility

Our findings reinforce a well-documented tension in psychiatric care: the marginalization of relational practices. Staff repeatedly described listening, talking, and being present as practices that “did not look like work,” echoing prior research showing that affective and relational labor is often invisible, under-acknowledged, and difficult to measure (e.g. Cutcliffe & Happell, 2009; Grant et al., 2015). Organizational valuation systems such as lean management and performance indicators often render certain forms of work visible and legitimate while obscuring others, particularly relational and affective dimensions of care (Timmermans & Berg, 2003; Zuiderent-Jerak & Berg, 2010; Boltanski & Thévenot, 2006; Johansson et al., 2010).

In our study, relational work remained largely invisible until it produced measurable outcomes, such as reductions in coercion, that could be recognized within the organizational logic of efficiency and documentation. This study extends that literature by showing how the legitimacy of relational work was renegotiated through the self-harm strategy. Initially dismissed as non-professional or “soft,” relational practices gained value when linked to visible system priorities, particularly reduced coercion. In this sense, relational work appeared to gain legitimacy not only through its intrinsic therapeutic value but through its translation into outcomes that aligned with organisational priorities, particularly reductions in coercion and conflict on the wards.

This finding resonates with research on the Safewards model (Bowers et al., 2014, 2015), which similarly identifies positive staff–patient relationships and mutual understanding as key mechanisms for reducing conflict and containment in psychiatric wards. In the Safewards trials, wards that implemented structured relational interventions—such as clear mutual expectations, reassurance, and consistent communication—achieved significant reductions in aggression and coercive measures (Bowers et al., 2014, 2015). The parallel between these findings and our study underscores that “being with patients” is not merely an act of compassion but an evidence-based component of safe and effective inpatient care.

## Patient Hierarchies and Moralization

The analysis also reveals how staff located patients within implicit hierarchies of legitimacy. Psychotic patients were understood as “truly ill” and without responsibility for their actions, whereas patients who self-harm were often framed as exercising choice and therefore accountable. This resonates with research documenting the moralization of self-harm (Favazza, 2011; Shaw, 2014; Lindgren et al., 2011) and the positioning of borderline personality disorder as a contested or “difficult” diagnosis (Ring & Lawn, 2019). This pattern aligns with recent work on structural stigma in borderline personality disorder, which shows how institutional practices, professional cultures, and diagnostic boundaries can reinforce unequal forms of legitimacy and care (Klein et al., 2022). These distinctions illustrate how staff drew implicit boundaries around what counted as legitimate psychiatric suffering. Behaviors interpreted as involuntary were more readily incorporated into psychiatry’s core mandate, whereas behaviors perceived as intentional or controllable risked being positioned at its margins. Notably, these distinctions persisted despite widespread professional knowledge that self-harm is closely associated with emotional dysregulation and psychiatric disorder (Jørgensen et al., 2024; Kongerslev et al., 2015), suggesting that moral interpretations of responsibility may coexist with, and sometimes override, clinical knowledge. This is particularly significant given strong evidence that targeted psychotherapies can lead to substantial reductions in self-harm and emotional instability (Storebø et al., 2022), underscoring that moral framings of self-harm as volitional have real clinical consequences.

Importantly, our findings illustrate how such hierarchies persisted even as staff adopted new strategies: relational care for self-harm patients remained precariously legitimized, vulnerable to moral judgments that positioned self-harm as outside “real psychiatry.” These distinctions also intersect with how psychiatric classification systems draw boundaries between mental illness and mental disorders, which can shape staff assumptions about volition, responsibility, and legitimacy (Klein et al., 2022).

## Cultural Change and Implementation Challenges

A central finding was that the self-harm strategy was experienced not simply as a new set of tools but as a cultural shift in inpatient practice. Staff contrasted earlier routines of coercion and control, searching for hidden objects or restraining “at any cost,” with the new approach of dialogue and acceptance. This transformation was unsettling, particularly in the early stages, when many perceived the strategy as cold-hearted or as signaling indifference to patient suffering.

In Lewin’s (1947) terms, this period of uncertainty reflects the “unfreezing” phase of organizational change, where established norms are disrupted before new ones stabilize. Staff were temporarily caught between two moral orders, control and dialogue, illustrating how culture change in healthcare requires both emotional and structural reorientation. From an institutional perspective, such shifts can also be viewed as institutional work (Cloutier et al., 2016), where frontline professionals enact change through everyday negotiations of meaning and legitimacy. Such resistance is consistent with research on complex interventions, which often require staff to challenge entrenched norms and reconfigure professional identities (Skivington et al., 2021).

For many, allowing patients to self-harm within clearly defined limits conflicted with their ethical and professional instincts as healthcare workers. The formal limit in the strategy concerned active suicide attempts, yet staff still needed to assess each situation in real time, since it was not always clear whether an episode of self-harm reflected suicidal intent, emotion regulation, or another function (Johal et al., 2025). This uncertainty contributed to the ethical tension they described.

Similar to findings by Johal et al. (2025), staff described the experience as “walking a tightrope” between preventing harm and respecting autonomy, often accompanied by feelings of discomfort and moral distress. Witnessing acts of self-harm, particularly cutting and the sight of blood, could evoke strong visceral and emotional reactions, reinforcing the impulse to intervene. Previous research has likewise shown that caring for individuals who self-harm or are suicidal evokes powerful emotions such as fear, guilt, and helplessness (Hagen et al., 2017), and that psychiatric nurses frequently experience moral distress when required to balance protection with respect for autonomy (Ohnishi et al., 2010; Walle et al., 2025). Similar emotional and ethical tensions have been reported across other professional groups in inpatient mental health care, suggesting that these reactions are not limited to nursing roles.

Over time, however, staff described a gradual legitimization of the new approach, driven by visible outcomes such as calmer inpatient environments and fewer coercive episodes. This aligns with findings from implementation science, where sustained change often depends on both experiential sense-making and demonstrable effects (Fixsen et al., 2005; Greenhalgh et al., 2017). Yet participants also pointed to structural barriers—including staff shortages, time pressures, and limited opportunities for reflection—that undermined the consistent application of the strategy. These barriers echo broader research on psychiatric inpatient care, which highlights how resource constraints and organizational demands erode the conditions needed for relational practices (Cutcliffe & Happell, 2009). A recent meta-synthesis similarly shows that clinicians working with adolescents who self-harm experience significant emotional strain and emphasize the need for structured spaces for reflection and support (Walle et al., 2025).

## Responsibility, Guilt, and Emotional Distance

The fourth theme highlights how staff negotiated responsibility for self-harm and the emotional costs this entailed. The new strategy required staff to refrain from coercive interventions unless harm was life-threatening, which many initially experienced as doing nothing or failing to protect the patient. This perception left some uncertain about professional boundaries and the meaning of their role. Feelings of guilt (“could I have done more?”) coexisted with efforts to maintain emotional distance, illustrating the ambivalence of staff positions. These accounts indicate that the strategy did not only demand emotional labour from staff but also required them to navigate ongoing moral uncertainty about responsibility and risk. Rather than simply managing emotions, staff described the challenge of acting within situations where the boundaries of professional responsibility were less clearly defined. Our analysis also connects to research on emotional labor, showing how psychiatric staff must simultaneously manage patients’ emotions and their own, a form of what Hochschild (2003) termed deep acting, in which professionals strive to genuinely feel composure and empathy in the face of distress. Such emotional regulation is both a professional requirement and a personal cost, often carried out without collective structures for support (Hochschild, 2003; Hagen et al., 2017).

Recognizing and supporting this aspect of practice is crucial for sustaining change and preventing burnout. Echoing findings from Boustani et al. (2025), who examined stress and supervision among paraprofessional mental health workers, our participants described how role ambiguity and limited structural support amplified emotional strain. Empirical evidence further highlights that structured clinical supervision, peer reflection, and organizational support can mitigate stress and strengthen professional resilience (Edwards et al., 2006; Boustani et al., 2025; West et al., 2016). Integrating such reflective and supportive structures across professional groups appears essential to maintain staff wellbeing and ensure the long-term viability of relational and dialogical approaches in self-harm care.

## Practical and Clinical Implications

The findings highlight several implications for clinical practice and organizational policy. First, there is a need to legitimize relational work as a core component of psychiatric care. In this context, relational work refers to the interpersonal and communicative practices used across professional groups, including supportive conversations, emotional attunement, and other forms of therapeutic engagement. Staff consistently described listening, talking, and being present as undervalued, yet these practices were central to preventing escalation and reducing coercion. Training programs should therefore emphasize not only technical tools but also the professional value of relational practices, framing them as skilled and active work rather than as “just talking.”

Second, the persistence of patient hierarchies indicates that staff may require support to critically reflect on assumptions that position some groups as more “deserving” of care than others. Interventions targeting self-harm should therefore explicitly address the moralization of patient behavior and create space for alternative understandings, such as self-harm as a coping strategy.

Third, the dilemmas of responsibility underscore the need for nuanced guidance and support. The self-harm strategy rightly emphasized moving away from paternalistic practices by encouraging patients to take an active role in understanding their behavior and adopting alternative strategies. Yet staff also described how this shift left them with feelings of guilt when patients continued to self-harm and a need to create emotional distance to cope with the strain. Guidance and supervision should therefore address not only how responsibility is shared with patients but also how staff can manage the emotional and moral burden of these dilemmas without becoming isolated or burnt out.

Fourth, the study underscores the importance of structural and organizational conditions. Staff pointed to time pressure, understaffing, and limited opportunities for reflection as barriers to enacting the strategy. These barriers also shaped how responsibility and guilt were managed: in the absence of structured supervision or reflection, staff often carried the emotional burden individually. Implementation efforts must therefore avoid placing the weight of cultural change solely on individual staff and instead create sustainable organizational support (Fixsen et al., 2005; Greenhalgh et al., 2017). Beyond adequate staffing, this includes embedding regular supervision and structured spaces for collective reflection, which are critical for making sense of unsettling changes and sustaining cultural transformation.

Finally, the findings suggest that visible outcomes such as coercion rates can play a crucial role in legitimizing interventions. While relational work remains difficult to measure directly, its value may be demonstrated indirectly through reductions in coercion, improved ward climate, and patient satisfaction. More broadly, these findings highlight that cultural change in psychiatric care requires both structural reinforcement and organizational learning (Lewin, 1947). Valuing relational work must therefore be built into the very mechanisms by which psychiatric organizations define and measure good care, recognizing that systems of documentation and evaluation actively shape what is seen as legitimate practice (Timmermans & Berg, 2003; Zuiderent-Jerak & Berg, 2010; Boltanski & Thévenot, 2006). Future research and policy should therefore prioritize outcome measures that capture the impact of relational practices, thereby securing their place on the clinical and political agenda.

## Study Limitations

There are several limitations to this study. The study was conducted in a single regional context, which may limit transferability to other psychiatric systems. Participants were self-selecting and may have been more engaged with the strategy than those who declined to participate. Finally, the researcher’s professional background in psychiatry shaped both sensitivity and potential bias in the analysis, although this was addressed through reflexive practices and critical dialogue within the research team. The strategy included structured supervision, educational activities, and increased organizational attention, which may themselves have supported cultural change and cannot be fully separated from the effects described by staff.

A strength of this study is the focus on staff perspectives across multiple professional groups, providing a rich and nuanced account of how a self-harm strategy was experienced in practice. The use of focus groups enabled participants to reflect collectively, revealing shared assumptions as well as tensions and disagreements. The reflexive thematic analysis approach allowed for an in-depth exploration of latent as well as semantic meanings, supported by reflexive memo-writing and team discussions.

The study’s reflexive and practice-oriented design aligns with the tradition of action research introduced by Lewin (1947), emphasizing inquiry as a means of supporting social and organizational learning. Although participants were not involved in verifying the transcripts or findings, credibility and dependability were supported through iterative team discussions, reflexive documentation, and transparent analytic decisions. These strategies strengthened the trustworthiness of the findings.

## Conclusion

This study shows that implementing a self-harm strategy in psychiatric inpatient care requires more than adopting new tools; it entails cultural change and reconfigured professional values. Staff described how relational practices, long marginalized, became gradually legitimized when linked to visible outcomes such as reduced coercion. More broadly, the findings suggest that cultural change in psychiatric care involves renegotiating both what counts as legitimate psychiatric suffering and what counts as legitimate psychiatric work. Patient hierarchies revealed enduring moral logics, positioning psychotic patients as “truly ill” and self-harming patients as responsible for their actions. The strategy also marked a cultural shift from control to dialogue, a transition that was initially resisted but increasingly accepted as its effects became evident. Finally, staff described the emotional and moral dilemmas of responsibility, including feelings of guilt, uncertainty about boundaries, and the need for emotional distance.

Sustaining self-harm strategies in everyday practice therefore depends on legitimizing relational care, creating collective spaces for reflection, and addressing the structural and emotional conditions that shape psychiatric work. Beyond psychiatry, these findings may also speak to wider debates in healthcare about how invisible or emotionally demanding aspects of care can be recognized and supported. (Table 1).

**Table 1:** Thematic map: Staff experiences of implementing the self-harm strategy

Theme	Subtheme	Illustrative quote
1. Relational work as invisible yet essential	Invisible work	“When I sit and just listen, it doesn’t look like I’m working – but that’s when things actually calm down.” (FG2)
	Sacrificed when busy	“The first thing we drop when we’re short on staff is the talking – because documentation and meds have to be done.” (FG1)
	Emotionally demanding	“It takes so much out of you, just being there with their pain.” (FG3)
	Legitimation through outcomes	“It only counts when they see fewer coercive episodes – then suddenly it’s real work.” (FG4)
	Relatives as barrier	“Families sometimes say: why are you just sitting there talking? They don’t see it as treatment.” (FG2)
2. Patient hierarchies – psychosis as legitimate psychiatry, self-harm as responsibility	Psychosis as legitimate	“If they’re psychotic, we know it’s not their fault – they’re really ill.” (FG1)
	Self-harm as choice / responsibility	“With self-harm, you feel they should be able to stop – like they’re in control.” (FG3)
	Outside psychiatry’s core	“It doesn’t feel like real psychiatry sometimes, more like social problems.” (FG2)
	Counter-narratives (coping)	“I try to see it as their way of surviving – not because they want to hurt us.” (FG4)
3. Cultural change – from control to dialogue	Earlier practice: control and coercion	“Before, we would search and restrain no matter what – that was just the routine.” (FG1)
	Resistance and unease	“At first it felt so wrong – like we were ignoring their suffering.” (FG2)
	Gradual legitimization through outcomes	“When we saw the number of belts go down, then people believed in it.” (FG3)
	Role of training and tools	“The crisis plans and joint language helped us stick with it.” (FG4)
	Work environment and emotional strain	“It’s easier when we have time to reflect – but when we’re under pressure, it slips.” (FG2)
4. Responsibility, guilt, and emotional distance	Ambiguous responsibility	“I think the hardest thing has been to stand there and watch someone harm themselves and not intervene, because it’s ingrained in you that you must stop it.” (FG3)
	Emotional burden and guilt	“It wears you down, that feeling that maybe you should have done more.” (FG3)
	Need for distance / self-protection	“I’m probably the one who runs the fastest to get away from it – sometimes you just can’t take it anymore.” (FG4)
	Uneven thresholds of tolerance	“It really depends on who you are as a person and how much you can take.” (FG4)
	Compassion fatigue	“When you keep giving that little extra, you reach a point where it’s too much – that’s when you start to shut down.” (FG3)

## Electronic Supplementary Material

Below is the link to the electronic supplementary material.

Supplementary Material 1

## Data Availability

The datasets generated and analyzed during the current study are not publicly available due to confidentiality agreements with participants but are available from the corresponding author on reasonable request. All data were stored on a secure, password-protected institutional server in accordance with the General Data Protection Regulation (GDPR) and Danish data-protection legislation.
