# Supplementary material for: It Doesn’t Look Like Work, but it Works: A Qualitative Study of Staff Experiences with a Self-Harm Strategy in Danish Inpatient Mental Health Services
Source: Adm Policy Ment Health. 2026 Apr 27;53(4):435–50. doi: 10.1007/s10488-026-01506-w (PMC13372923; doi:10.1007/s10488-026-01506-w)
Supplement: Supplementary file 1 — Supplementary Material 1 [file 10488_2026_1506_MOESM1_ESM.docx]

**Focus Groups on Staff Experiences With the Self-Harm Strategy**

**Content**

• Introduction by Kristine (10 minutes)
• Brief round of introductions (15 minutes)
• Group dialogue based on five questions about the impact of implementing the Self-Harm Strategy (55 minutes plus a 20-minute break)
• Summary and final reflections (15 minutes): Is there anything we have not yet covered?
• Closing (20 minutes): How was it for you to participate in the focus group?

**Presenting the Project**

A short introduction about me and the background of the project.

The implementation of Region Zealand’s Self-Harm Strategy is based on ten principles developed by an expert group. These principles draw on theoretical frameworks and methods with evidence for reducing self-harming behavior in outpatient therapeutic settings. We still lack knowledge on whether these concepts work as part of an overarching strategy in inpatient wards, where the structure and therapeutic frame differ from outpatient treatment. Region Zealand’s Mental Health Services adopted the strategy in spring 2022 as a concept with clear procedural guidelines for psychiatric staff. The strategy also contains theoretical assumptions and logics that the region works from.

There has been interest in how evidence-based methods affect patients, but we have limited knowledge about how they affect practitioners, meaning you. I am therefore interested in how staff have received the Self-Harm Strategy and the teaching, training, and supervision that followed, and how it has influenced your everyday work with the principles.

**Purpose of the Focus Groups**

A focus group interview differs from a traditional interview. You will be speaking the most, and I will invite you to discuss topics with each other, one at a time. You are welcome to express agreement or disagreement with what others say, and you may comment on each other’s reflections. There are no right or wrong answers and no correct or incorrect interpretations of my questions. I am here to learn. You are the experts. I am interested in your experiences rather than your factual knowledge.

**Agenda for the Meeting**

**Duration:** 150 minutes plus lunch
**Location:** Poppelhus, 2nd floor, Research Unit East, Conference Room

1. Introduction by the moderator
2. Round of introductions
3. Group dialogue
4. Summary
5. Closing

**1) Introduction by the Moderator (10 minutes including practicalities)**

• Group guidelines: confidentiality, anonymity, and respect for different viewpoints
• Only one person speaks at a time so everyone can be heard clearly
• You are welcome to take notes if there is something you want to remember to mention
• We are interested in your different experiences and perspectives on the Self-Harm Strategy
• Please think about your reflections in relation to two time points:
– Spring 2022, when the Regional Self-Harm Strategy was adopted
– Spring 2023, when you attended the training day on self-harm and the subsequent supervision
• I would like the focus to be on you and not on the person you may have lost
• There are no right or wrong answers
• We encourage everyone to participate so we can include as many perspectives and nuances as possible
• The interview will be audio recorded. Data will be handled confidentially and used only for research
• This is one of four focus groups. An article will be published based on the findings
• All data will be anonymized. No one will be able to identify you or those you talk about, although you may recognize your own statements if they are used as quotations
• We hope the knowledge gained will help identify what is helpful or unhelpful for inpatient staff when working with self-harm
• The session will last a maximum of 150 minutes. We begin with introductions, then the dialogue, and end with a closing round
• Feel free to get up and take coffee or tea
• Any questions before we begin?

**2) Round of Introductions (maximum 15 minutes)**

Please introduce yourselves by writing down and handing the following to Lisbeth:
• First name
• Ward or unit
• Age
• Profession
• How long you have worked in Region Zealand East

A short break is possible before we start the dialogue.

**3) Group Dialogue (55 minutes plus a 20-minute break)**

Here are five questions about your experiences with the Self-Harm Strategy and the training you received.

Please reflect on your answers in relation to the two time points: 2022 (adoption of the strategy) and 2023 (rollout of the training).

First, please agree on the order in which you want to discuss the questions.
You are also responsible for keeping track of time so all questions are covered.

**1) How have you experienced the implementation of the Regional Self-Harm Strategy?**

This is an open question about your experience of the organizational change in 2022.

**2) What have you taken from the teaching and supervision related to self-harm?**

This focuses on the period beginning with the training in May or June 2023 and the targeted efforts that followed.

**3) How do coercive situations involving patients who self-harm differ before and after the Self-Harm Strategy?**

This explores whether you have noticed changes and how you would describe them.
The question focuses directly on the outcome of coercion.

**4) In what ways has your understanding of working with patients who self-harm changed?**

This addresses whether staff sense a shift in their knowledge or attitudes and relates to the hypothesis that attitudinal change may mediate practice.

**5) What barriers do you experience when working with self-harm in inpatient wards?**

This concerns perceived challenges, including structural or organizational obstacles.

**Moderator prompts may include:**
“What do others think when you hear this?”
“Do any of you have a similar experience?”
“So now we hear another nuance or perspective.”

The moderator may also ask about exceptions to support critical reflection. For example:
“You highlight that having time to talk is important. Have you experienced situations where that was not possible? What did that mean for you?”

**4) Summary (15 minutes)**

We now move to the summary. Before we close, I would like to hear whether anything important has not yet been mentioned. This includes anything necessary to understand what is helpful or unhelpful for you when working with the Self-Harm Strategy and how the teaching and supervision have functioned.

**5) Closing (20 minutes)**

Thank you for sharing your experiences. I would like to hear how it has been for you to take part in the focus group.

If you think of anything in the coming days, you are welcome to send it to me by email.
